# Conditioned Media Derived from Human Adipose Tissue Mesenchymal Stromal Cells Improves Primary Hepatocyte Maintenance 

**DOI:** 10.22074/cellj.2018.5288

**Published:** 2018-05-28

**Authors:** Zahra Azhdari Tafti, Mehdi Mahmoodi, Mohamad Reza Hajizadeh, Vahid Ezzatizadeh, Hossein Baharvand, Massoud Vosough, Abbas Piryaei

**Affiliations:** 1 Department of Stem Cells and Developmental Biology, Cell Science Research Center, Royan Institute for Stem Cell Biology and Technology, ACECR, Tehran, Iran; 2 Department of Clinical Biochemistry, School of Medicine, Rafsanjan University of Medical Sciences, Rafsanjan, Iran; 3 Molecular Medicine Research Center, Rafsanjan University of Medical Sciences, Rafsanjan, Iran; 4 Department of Medical Genetics, Medical Laboratory Center, Royesh Medical Group, Tehran, Iran; 5 Department of Developmental Biology, University of Science and Culture, Tehran, Iran; 6 Department of Regenerative Biomedicine, Cell Science Research Center, Royan Institute for Stem Cell Biology and Technology, ACECR, Tehran, Iran; 7 Department of Biology and Anatomical Sciences, School of Medicine, Shahid Beheshti University of Medical Sciences, Tehran, Iran; 8 Department of Tissue Engineering and Applied Cell Sciences, School of Advanced Technologies in Medicine, Shahid Beheshti University of Medical Sciences, Tehran, Iran

**Keywords:** Conditioned Medium, Mesenchymal Stromal Cell, Primary Hepatocyte, Regenerative Medicine

## Abstract

**Objective:**

Recent advances in cell therapy have encouraged researchers to provide an alternative for treatment and restoration of damaged liver through using hepatocytes. However, these cells quickly lose their functional capabilities in vitro. Here, we aim to use the secretome of mesenchymal stromal cells (MSCs) to improve in vitro maintenance conditions for hepatocytes.

**Materials and Methods:**

In this experimental study, following serum deprivation, human adipose tissue-derived MSCs (hAT-MSCs) were cultured for 24 hours under normoxic (N) and hypoxic (H) conditions. Their conditioned media (CM) were subsequently collected and labeled as N-CM (normoxia) and H-CM (hypoxia). Murine hepatocytes were isolated by perfusion of mouse liver with collagenase, and were cultured in hepatocyte basal (William’s) medium supplemented with 4% N-CM or H-CM. Untreated William’s and hepatocyte-specific media (HepZYM) were used as controls. Finally, we evaluated the survival and proliferation rates, as well as functionality and hepatocyte-specific gene expressions of the cells.

**Results:**

We observed a significant increase in viability of hepatocytes in the presence of N-CM and H-CM compared to HepZYM on day 5, as indicated by MTS (3-(4,5-dimethylthiazol-2-yl)-5-(3-carboxymethoxyphenyl)-2-(4-sulfophenyl)- 2H-tetrazolium) assay. Indocyanine green (ICG) uptake of hepatocytes in the H-CM and HepZYM groups on days 3 and 5 also suggested that H-CM maintained the hepatocytes at about the same level as the hepatocyte-specific medium. The HepZYM group had significantly higher levels of albumin (Alb) and urea secretion compared to the other groups (P<0.0001). However, there were no significant differences in cytochrome activity and cytochrome gene expression profiles among these groups. Finally, we found a slightly, but not significantly higher concentration of vascular endothelial growth factor (VEGF) in the H-CM group compared to the N-CM group (P=0.063).

**Conclusion:**

The enrichment of William’s basal medium with 4% hAT-MSC-H-CM improved some physiologic parameters in a primary hepatocyte culture.

## Introduction

The liver is a vital organ in the body that regulates metabolism and maintains homeostasis. Hepatocytes are specific liver parenchymal cells that have high restoration and regeneration capacities (1, 2). Although orthotopic liver transplantation (OLT) is the gold standard treatment for end- stage patients, this approach is restricted due to obstacles such as the limited source of compatible donated organs, rejection by the recipient’s immune system, and high cost (3, 4). Considering these limitations, cell replacement therapy is currently used as an alternative treatment method for organ transplantation (3, 5). Researchers have injected primary hepatocytes intravenously from a compatible healthy donor into respective patients and have observed that the transplanted cells could successfully migrate into the liver parenchyma, repair the damaged tissue, and restore the function of this organ (6, 7).

In recent years, several investigations have attempted to differentiate various types of stem cells into hepatocyte-like cells (8-10). This approach could potentially culminate the application of the hepatocyte-like cells in the clinic, as well as drug discovery and toxicology studies (11). However, due to the lack of cell-matrix and cell-cell interactions, hepatocytes and hepatocyte-like cells quickly dedifferentiate in vitro and lose their distinctive properties (12). Therefore, finding an approach to preserve the potency of these differentiated cells is indispensable.

It has been proposed that mesenchymal stromal cells (MSCs) could be beneficial for the recovery and regeneration of liver tissue. MSC transplantation in several preclinical studies or human patients revealed satisfactory outcomes with regards to liver regeneration, most likely due to the biochemical factors derived from MSCs (2, 3). The liver trophic factors secreted by MSCs, particularly hepatocyte growth factor (HGF) could be the crucial player in liver regeneration (13, 14). Therefore, recent studies have used the secreted factors rather than direct application of MSCs (15).

It has been shown in a previous study that the secretome collected from MSC cultures could remarkably improve the survival rate of animals with acute liver failure, reducing hepatic cell death and stimulating hepatocyte proliferation up to three folds (16). Other researchers have suggested that MSCs co-cultured with hepatocytes could elevate the level of albumin (Alb) secretion as the number of apoptotic hepatocytes decline (12, 17).

Some of the biochemical factors that are expressed in MSCs, such as HGF, epidermal growth factor (EGF), interleukin (IL)-6, vascular endothelial growth factor (VEGF) and insulin-like growth factor binding protein (IGFBP), could prohibit hepatocyte apoptosis after liver injuries (16, 18).

Therefore, these findings have turned the application of cytokines, growth factors and other biochemical factors obtained from MSC cultures into an optimal strategy, compared to the use of the actual MSCs, for maintenance of hepatocytes (17, 19).

Among different types of MSCs, the adipose tissue MSCs (AT-MSCs) are a superior option compared to bone marrow MSCs (BM-MSCS) with regards to their feasibility in isolation, access to autologous sources with less invasive methods (20), as well as higher levels of HGF and VEGF production (19, 21-23). Since less concentration of oxygen is required to grow these cells in an appropriate niche (24), preconditioning of AT-MSCs with hypoxia (1-3% oxygen) can change aerobic metabolism into anaerobic metabolism and induce the secretions of VEGF, HGF, IL-6, EGF, and erythropoietin (25, 26). This condition could also increase the cell survival rate by activating Akt, c-Met and cyclin-D1, which play crucial roles as the HGF receptor and in cell cycling (27, 28).

In this study, we investigated the effects of AT-MSC secreted factors, obtained from the MSC cultures, on hepatocyte maintenance *in vitro*. We found that the presence of factors from hAT-MSCs in primary hepatocyte cultures promoted their proliferation rate and accelerated some of their specific functions.

## Materials and Methods

In this experimental study, there were three independent Azhdari Tafti et al. biological repeats for all experiments. All procedures in our studies were monitored and approved approved at Royan Ethics Committee under the approval code EC/93/1031.

### Human adipose tissue-derived mesenchymal stromal cell culture

The hAT-MSCs obtained from Royan Institute Stem Cell Bank (Tehran, Iran). All the cells in this bank are donated by donors who have signed informed consents. MSC medium consisted of Dulbecco’s Modified Eagle’s Medium low glucose (DMEM-LG, Gibco, USA), supplemented with 10% fetal bovine serum (FBS, Gibco, Mexico), 1% penicillin- streptomycin, and 2% L-glutamine (Gibco, Japan). MSCs were incubated at 37°C in a 5% humidified CO2 chamber, to reach 80% confluency, while the medium was replaced with fresh medium every three days. These cells were cultured for three passages, followed by characterization using different techniques based on the International Society for Cellular Therapy (ISCT) guidelines, including flow cytometry and directed differentiation into bone and adipocyte. 

### Characterization of human adipose tissue-derived mesenchymal stromal cells

We used passage-3 cells for immuno-phenotype analyses. The cells were washed with phosphate-buffered saline (PBS, Gibco, USA) and dissociated enzymatically with 0.5% trypsin (Gibco, USA). Following another PBS wash these cells were blocked with 2% FBS/PBS for 30 minutes at 37°C. Next, the cells were incubated with anti-CD45-FITC/CD34-PE, antiCD73- PE (BD, USA), anti-CD90-FITC (Dako, USA), and anti-CD105-PE (R&D Systems, USA) antibodies for 1 hour at 4°C. A specific isotype control (mouse IgG1-FITC/PE, Dako, USA) was utilized to determine background staining. Three independent biological experiments were carried out for the individual markers, and the data were analyzed using the CellQuest™ program (BD FACSCalibur, USA). 

To evaluate the multilineage differentiation potential of hAT-MSCs the cells were seeded (30000 cells/well) in 6-well plates. Once the cells reached 80% confluency we added either adipogenic medium [DMEM, supplemented with 50 µg/mL ascorbic acid 3-phosphate, 100 nM dexamethasone, 50 µg/mL indomethacin (all from Sigma-Aldrich, USA)] or osteogenic medium [DMEM, supplemented with 50 µg/mL ascorbic acid 2-phosphate, 10 nM dexamethasone, 10 mM ß-glycerol phosphate (all from Sigma-Aldrich, USA)] to the culture. The media were refreshed twice per week. After three weeks of differentiation, adipogenesis and osteogenesis were evaluated by oil red-O and alizarin red staining, respectively, according to standard protocols. Furthermore, the samples were collected for specific gene expression analysis using reverse transcription polymerase chain reaction (RT-PCR). 

### Condition medium preparation

After characterization, passage-4 hAT-MSCs were divided into two groups and cultured until 70% confluency. Then, the medium was discarded and the cells were washed twice with PBS, followed by the addition of DMEM-LG, supplemented with 0.1% human serum albumin (HAS, Aventis, Germany). One group of MSCs was treated with 5% oxygen (hypoxia, H), while the other group was treated with21% oxygen (normoxia, N). After 24 hours, culture mediawere collected from both groups and centrifuged at 2500rpm for 10 minutes at 4°C. Supernatants were subsequentlyconcentrated up to 24 folds by Amicon Ultra-15, 3kDa cutoff Centrifugal Filter Unit (EMD Millipore, Ireland).

### Mouse hepatocyte isolation and culture

Male NMRI mice (6-8 weeks old) were anesthetized by intraperitoneal injections of 80 mg/kg ketamine and8 mg/kg xylazine (Alfasan, The Netherlands). We used aHepatocyte Isolation System (Worthington Kit, USA) forliver perfusion. Briefly, pre-warmed (37°C) Hanks balancedsalt solution (HBSS) supplemented with ethylene glycol- bis(2-aminoethylether)-N,N,N,N, tetraacetic acid (EGTA, Sigma, USA) was perfused (6 mL/minute) into the livertissue through the portal vein. Subsequently, collagenase- DNase digestion enzymes were perfused and circulated inthe liver tissue for 5-6 minutes. Then, the mice euthanized by dissecting the liver out and the softened liver tissue weretransferred to a sterile tube that contained 15 mL of cold Leibovitz’s L-15 Medium (Gibco, USA). Hepatocytes weredispersed into single cells by pipetting. Suspended cells weresubsequently passed through a sterile filter mesh (70 µm), transferred to 25 mL ice cold William’s medium E (Sigma, USA) that contained 2% penicillin-streptomycin, and centrifuged at 2500 rpm for 3 minutes at 4°C. Cell pelletswere collected, washed twice in PBS, and counted by 0.4% trypan blue (Merck, Germany) staining method. 

**Fig.1:**
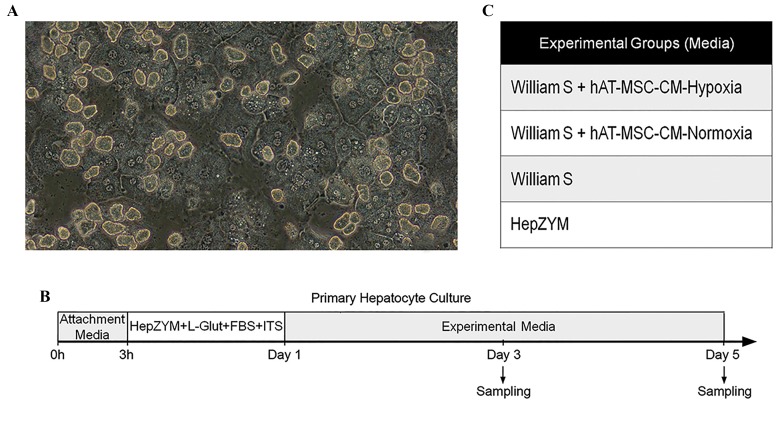
Mouse primary hepatocytes in culture. A. Representative image of mouse primary hepatocyte morphology (scale bar: 100 µm), B. Hepatocytes cultured in four different media, and C. Schematic protocol of primary hepatocyte culture and sampling time points. hAT-MSC-CM; Human adipose tissue-mesenchymal stromal cells-conditioned media, FBS; Fetal bovine serum, and ITS; Insulin, transferrin, selenium.

Hepatocytes were then re-suspended in attachment medium (William’s medium, supplemented with 5% FBS, 1% L-glutamate, and 2% penicillin-streptomycin) and plated in matrigel-coated wells at 50,000 hepatocytes/cm^2^ (Fig .1A). After 3 hours, the medium was replaced with Hepatozym- SFM (HepZYM, Sigma-Aldrich, USA) that contained 20% FBS, 1% L-glutamate, 1% insulin (ITS, Gibco, USA) and 2% penicillin-streptomycin for 21 hours (Fig .1B).

We divided the cultured hepatocytes into 4 experimental groups. Each group was treated with HepZYM medium, William’s medium, William’s medium supplemented with 4% N-CM or William’s medium supplemented with 4% H-CM (Fig .1C). The samples were harvested on days 3 and 5. 

### Gene expression analysis

Total RNA was isolated from passage-4 MSCs as well as MSCs cultured in either adipogenic or osteogenicmedium, using TRIzol (Ambion, USA) according to the manufacturer’s instructions. Furthermore, total RNA from hepatocytes cultured in the four groups of experimental media on days 3 and 5 were extracted by MN Nucleospin RNAII (MACHEREY-NAGEL, Germany). Then, 1 µg of total RNA was utilized to make cDNA with the Fermentas kit (Thermo Fisher Scientific, Germany) for RNA derived from MSCs and the Prime Script™ RT Reagent Kit (TaKaRa, Japan) for hepatocyte RNA. Subsequently, reverse transcription-polymerase chain reaction (RTPCR) and gel electrophoresis were performed to evaluate MSC-multilineage potential, and quantitative RT-PCR (qRT-PCR) for hepatocyte gene expression, using specific primer sets (Table 1). Endogenous housekeeping genes for RT-PCR (*ß-ACTIN*) and qRT-PCR (*Gapdh*) were used as the reference genes. The q-RT-PCR assay was performed using cDNA power SYBR green (TaKaRa Clonetech, Japan). The reaction was carried out in three independent biological experiments using a real-time PCR machine (Corbett Life Science, Qiagen, USA). Relative quantification was determined using the 2^-ΔΔCt^ method. 

Primers used to characterize hAT-MSCs in conventional RT-PCR or hepatocyte gene expression by qRT-PCR are listed in Table 1.

**Table 1:** Primers used to characterize hAT-MSCs in conventional RT-PCR or hepatocyte gene expression by qRT-PCR


	Target gene	Primer sequence (5´-3´)	Accession number	Product length (bp)

Genes for MSC multi-lineage potential	*β-ACTIN*	F: TCCCTGGAGAAGAGCTACG	NM_ 001101.3	131
		R: GTAGTTCGTGGATGCCACA		
	*GBP28*	F:CCTGGTGAGAAGGGTGAGAA	NM_001177800.1	174
		R:CAATCCCACACTGAATGCTG		
	*LPLl*	F: TCAACTGGATGGAGGAGGAG	NM-001177800-1	169
		R: GGGGCTTCTGCATACTCAAA		
	*OCN*	F: GTG CAG AGT CCA GCA AAG GT	NM_000088.5	175
		R: TCA GCC AAC TCG TCA CAG TC		
	*COL1A1*	F: ATGCCTGGTGAACGTGGT	NM_000088.3	87
		R: AGGAGAGCCATCAGCACCT		
Specific genes for hepatocytes	*Gapdh*	F: GACTTCAACAGCAACTCCCAC	NM_008084	125
		R: TCCACCACCCTGTTGCTGTA		
	*Alb*	F: AGA CAT CCT TAT TTC TAT GCC C	NM_009654.3	141
		R: GAC CAA TGC TTT CTC CTT CAC		
	*Cyp2b9*	F:CTGGCCACCATGAAAGAGTT	NM_010000.2	153
		R:GATGATGTTGGCTGTGATGC		


hAT-MSC; Human adipose tissue-mesenchymal stromal cells and qRT-PCR; Quantitative real time-polymerase chain reaction.

### Hepatocyte viability and proliferation assay

 We used 3-(4,5-dimethylthiazol-2-yl)-5-(3carboxymethoxyphenyl- 2-(4-sulfophenyl)-2Htetrazolium) (MTS) assay (Promega, USA) to evaluate the viability and proliferation rates of the hepatocytes treated with the CMs (N-CM and H-CM) on days 3 and 5, according to manufacturer’s instruction. Briefly, 50000 primary hepatocytes/cm^2^ were cultured in a 24-well cell culture plate (TPP, Switzerland) using the assigned medium for each experimental group. At the respective time, the medium was removed and 200 µL William’s medium containing 40 µL of MTS assay solution were added to each wells and incubated at 37°C for 90 minutes. Subsequently, the absorbance of the incubated medium was detected at 490 nm, and normalized to a blank sample (William’s medium in presence of hepatocytes), using ELISA microplate reader (Thermo Scientific, USA). Each experiment was technically performed in triplicate. 

### Glycogen storage assay

Glycogen storage was evaluated by periodic acid-Schiff (PAS) staining on days 3 and 5. We quantified the PAS- positive areas by ImageJ software (Version 1.46 developed at NIH; https://rsb.info.nih.gov/ij/). A total of 5 random fields per sample (15 per group, n=3) were independently analyzed.

### Indocyanine green uptake

Indocyanine green (ICG) uptake was evaluated by incubating the cells for 30 minutes in a mixture of 30 µL ICG (CardioGreen, Sigma-Aldrich, USA) and 320 µL William’s medium. Quantification of this test was the same as the PAS analysis with ImageJ software.

### Cytochrome P450 activity 

Hepatocyte function was evaluated for Cyp2b9 cytochrome P450 activity by the pentoxyresorufin o-dealkylase (PROD) test. Briefly, 5×10^4^ hepatocytes/cm^2^ were cultured for 3 or 5 days using the assigned medium for each group. Next, the medium was removed, the cells were washed with PBS, and subsequently incubated in 300 µL HBSS medium containing 1 µL of 7-pentoxyresorufin (5 µM dissolved in DMSO, Sigma-Aldrich, USA) as well as 2.5 µL dicumarol (Sigma-Aldrich, USA) for 30 minutes at 37°C in the dark. Later, we collected the supernatant in order to determine fluorescent intensity at 830-890 nm using a Synergy4 microplate reader (BioTek, USA). The values were normalized to the negative control, hepatocytes cultured in HBSS medium without 7-pentoxyresorufin and dicumarol.

### Albumin and urea production 

Hepatocyte conditioned media (CM) were collected on days 3 and 5 from the different groups. We evaluated Alb production using a Mouse Albumin ELISA Quantitation Kit (Bethyl Laboratories, USA) and urea secretion with a Urea Assay Kit (Biorex, UK). The values were normalized to the total protein acquired from a Total Protein Kit (Biorex, UK) that used an auto analyzer (Alpha-Classic, Iran).

### Evaluation of secreted growth factors in the conditioned media

We evaluated the presence and the amount of hepatocyte-supporting growth factors, VEGF, HGF and basic fibroblast growth factor (bFGF), that were secreted by hAT-MSCs into the CMs under normoxic and hypoxic conditions after 2 days in culture. The growth factors were evaluated by commercially available ELISA kits (R&D Systems, USA) according to the manufacturer’s protocols. The experiments were technically repeated twice. 

### Statistical analysis

Statistical analyses were performed using SPSS, version 21. Data were presented as mean ± SD. Measurements were carried out using analysis of variance (ANOVA) and we chose the LSD method for post hoc multiple comparisons. AP value of 0.05 was considered significant. All graphs were delineated by Graphpad-prism, version 6.

## Results

### Characterization of human adipose tissue-derived mesenchymal stromal cells 

**Fig.2:**
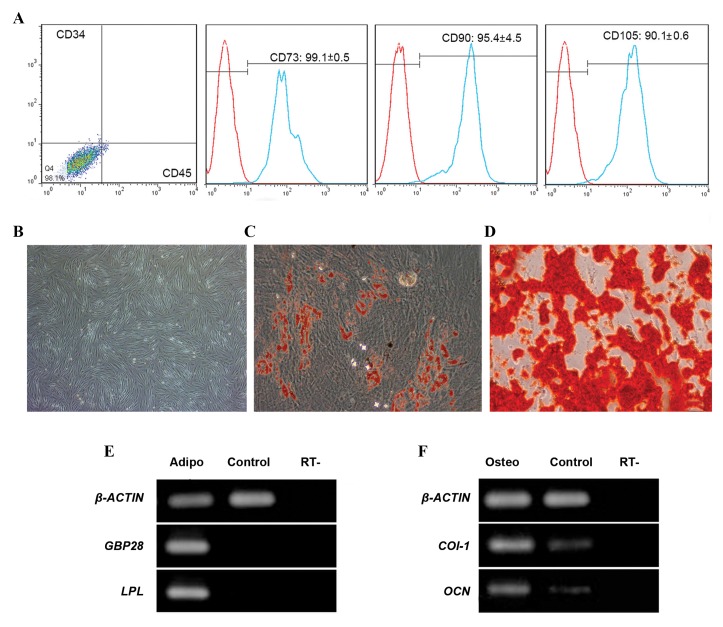
Characterization of human adipose tissue mesenchymal stromal cell (hAT-MSCs) after three passages. A. Flow cytometric analysis for MSC surface markers (CD73, 90 and 105) and hematopoietic markers (CD34 and CD45). Representative images of B. MSCs cultured in (scale bar: 100 µm) C. Adipogenic (scale bar: 100 µm), D. Osteogenic media for three weeks, followed by staining with oil red O for adipocytes and alizarin red for osteocytes (scale bar: 50 µm). Reverse transcription-polymerase chain reaction (RT-PCR) analysis demonstrated expression of E. Adipogenic (*LPL, GBP28*) and F. Osteogenic (*COL1, OCN*) genes, in differentiated cells cultured in their respective media for three weeks. *ß-ACTIN*; Internal control, and Control; hAT-MSC.

Flow cytometry analysis confirmed the expressions of the mesenchymal surface markers CD73, CD90, and CD105 (Fig .2A) in the cutured hAT-MSCs. These cells did not express the hematopoietic surface markers, CD34 and CD45. hAT-MSCs showed spindle-like fibroblast shape at the 3^rd^ passage (Fig .2B). We used the appropriate differentiation protocols to differentiate MSCs into osteocytes and adipocytes. Oil red-O staining showed an accumulation of lipid droplets in the adipocytes derived from MSCs (Fig .2C), while alizarin red staining revealed mineralized nodules in the periphery of the generated osteocytes (Fig .2D). RT-PCR analysis confirmed that the differentiated cells in the adipogenic medium expressed adiponectin (*GBP28*) and lipoprotein lipase (*LPL*) genes (Fig .2E). Collagen type 1 (*COL-1*) and osteonectin (*OCN*) genes were expressed in the differentiated cells under osteogenic conditions (Fig .2F). 

### hAT-MSCs conditioned medium improved viability and hepatocyte proliferation 

The cultured hepatocytes showed a cuboidal morphology with polyploidy (Fig .1A). The experimental groups and study overview are presented in Figure 1B and C.

**Fig.3:**
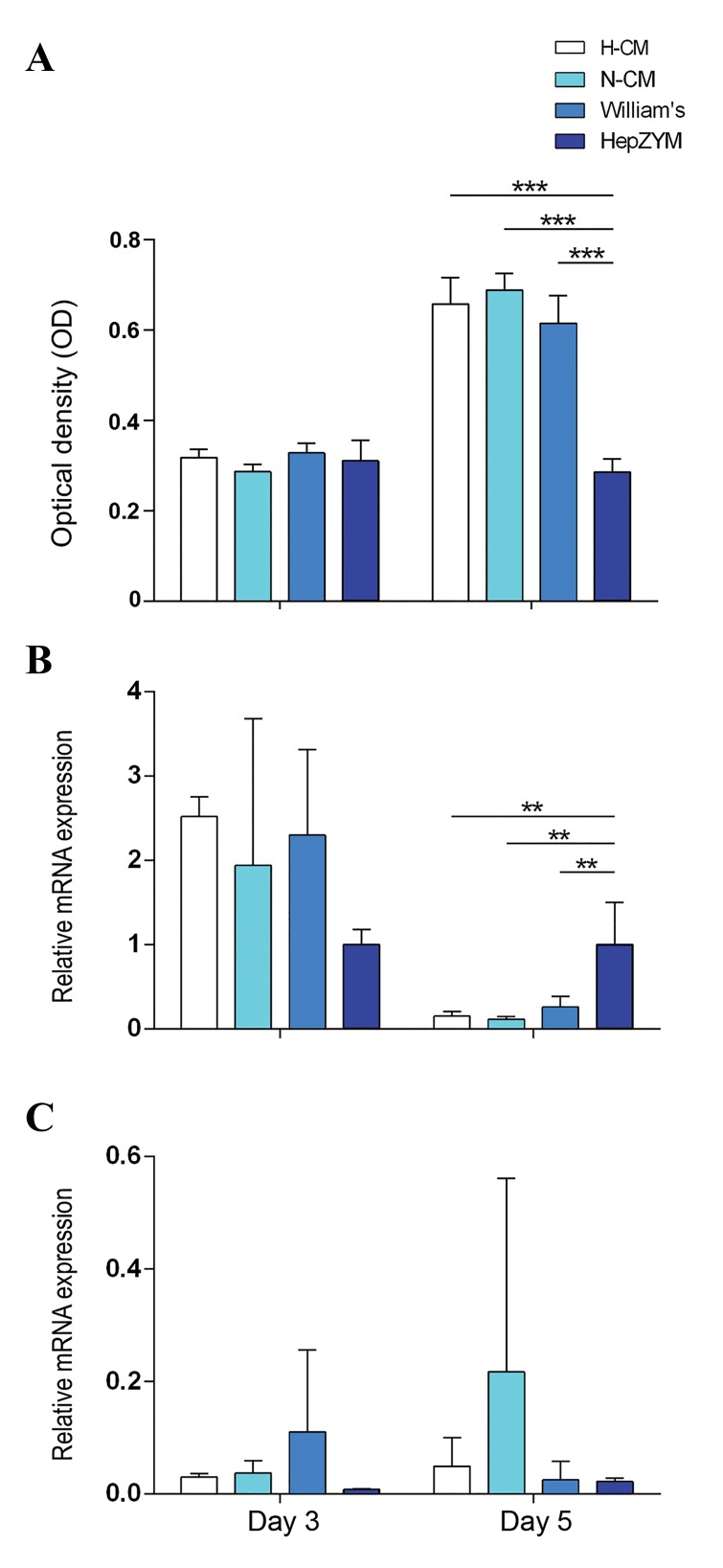
Hepatocyte viability and relative expression of Alb and Cyp2b9 in different conditioned mediua after 3 or 5 days of culture. A. Cell viability analysis using MTS assay. On day five, the viability of hepatocytes were significantly higher in H-CM, N-CM and William’s medium (P=0.0001) compare to HepZYM. B and C. mRNA expression of Alb and Cyp2b9 genes using qRT-PCR. The values were normalized to Gapdh, as the housekeeping gene. The Alb expression on day 5, in H-CM, N-CM and William’s groups were significantly reduced compared to HepZYM. The P-values for all comparisons were 0.001. The data were presented as mean ± SD (n=3, **; P<0.001, and ***; P<0.0001). H-CM; hypoxic-conditioned media, N-CM; Normoxic-CM, and q-RT-PCR; Quantitative real time-polymerase chain reaction.

We evaluated the effects of the CMs on maintenance of primary hepatocytes. No significant difference existed afterthree days of culture in the MTS assay; however, on day 5optical density (OD) significantly increased in culture mediasupplemented with N-CM, H-CM, and William’s mediumcompared to the cells cultured in HepZYM (Fig .3A). Itmeans that the viability or proliferation of hepatocytes wassignificantly higher in H-CM, N-CM and William’s medium(P=0.0001) compare to HepZYM on day 5. The rise of ODin 5^th^ day compared to 3^rd^ day suggested that the secretomeof hAT-MSC stimulate proliferation in primary hepatocytes *in vitro*. We observed no significant difference between Nor H hAT-MSCs-CM and William medium in terms of cell viability and proliferation. 

### *Alb* and *Cyp2b9* expressions

We assessed the maintenance of primary hepatocytes in the presence of CMs by qRT-PCR to measure the relative expressions of *Alb* and *Cyp2b9* on days 3 and 5. The data showed no significant differences in *Alb* or *Cyp2b9* expression in different groups after 3 days of culture (Fig .3B, C). Further analysis, however, showed that *Alb* expression significantly decreased (P=0.001) after 5 days in all groups in comparison to the group incubated in HepZYM medium (Fig .3B), which could be due to de-differentiation of the primary hepatocytes in culture after 5 days.

### hAT-MSCs conditioned medium supported glycogen storage on day 3

**Fig.4:**
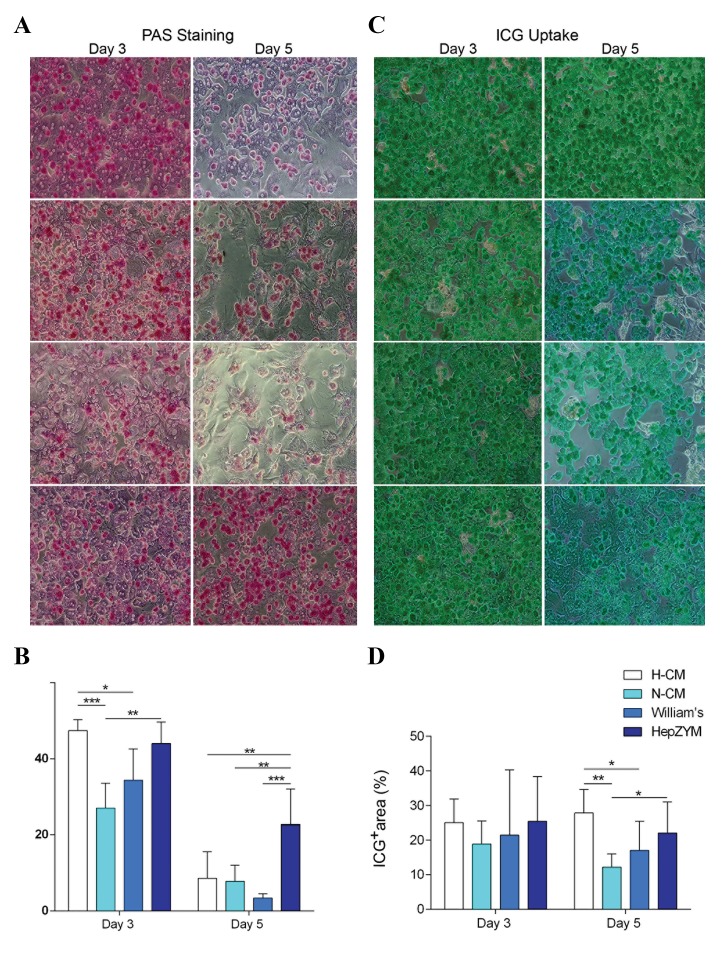
Liver-specific function analysis of hepatocytes in different media on days 3 and day 5. A, B. Representative images and quantitative analysis of PAS staining for cultured hepatocytes. On day 3, the PAS^+^ areas in H-CM significantly increased, compared to N-CM (P=0.0001) and William’s medium (P=0.021). The PAS^+^ areas in N-CM were significantly (P=0.004) less than HepZYM. Furthermore, the PAS^+^ areas in HepZYM were significantly higher than H-CM and N-CM (P=0.001 for both) and also William’s medium (P=0.0001), C and D. Representative images and quantitative analysis for indocyanine green (ICG)-uptake in hepatocytes. There was no significant difference in ICG uptake on day 3 in different groups. On day 5, the ICG uptake in H-CM was significantly higher than N-CM (P=0.001) and William’s medium (P=0.017). The ICG uptake in HepZYM group was significantly (P=0.012) higher than N-CM group. The data were presented as mean ± SD (n=5, *; P<0.05, **; P<0.001, and ***; P<0.0001) (scale bar: 100 µm). PAS; Periodic acid-Schiff, H-CM; hypoxic- conditioned media, N-CM; Normoxic-CM, and hAT-MSC-CM; Human adipose tissue-mesenchymal stromal cells- conditioned media.

In this study, we evaluated the effects of hAT-MSC-CMson glycogen storage as one of the characteristic features ofhepatocytes (Fig .4A). The percentage of PAS^+^ areas in the H-CM treated group was similar to the HepZYM group, butsignificantly higher than the N-CM (P=0.0001) and William’s(P=0.021) groups on day 3 of cell culture (Fig .4B). However, the PAS^+^ areas in N-CM were significantly (P=0.004) lessthan in HepZYM. On day 5, there was a reduction in the PAS^+^ areas in all groups. However, HepZYM-treated hepatocytesshowed significantly more glycogen storage capabilitycompared to the other groups. The PAS^+^ areas in HepZYMwere significantly higher than the cells in H-CM and N-CM(P=0.001 for both) on day 5. Furthermore, the PAS^+^ areas in William’s medium were significantly (P=0.0001) less than HepZYM group.

### hAT-MSCs conditioned medium protects indocyanine green uptake

We evaluated the level of ICG uptake in the hepatocytes(Fig .4C). The findings showed that ICG uptake in theH-CM treated group was similar to the HepZYM group, but significantly was higher in H-CM group compared toN-CM (P=0.001) and William’s medium (P=0.017) on day 5. Furthermore, on day 5 the ICG uptake in HepZYM group was significantly higher (P=0.012) than the N-CM group. There was no significant difference in ICG uptake on day 3 in different groups (Fig .4D).

### Cytochrome P450 activity

**Fig.5:**
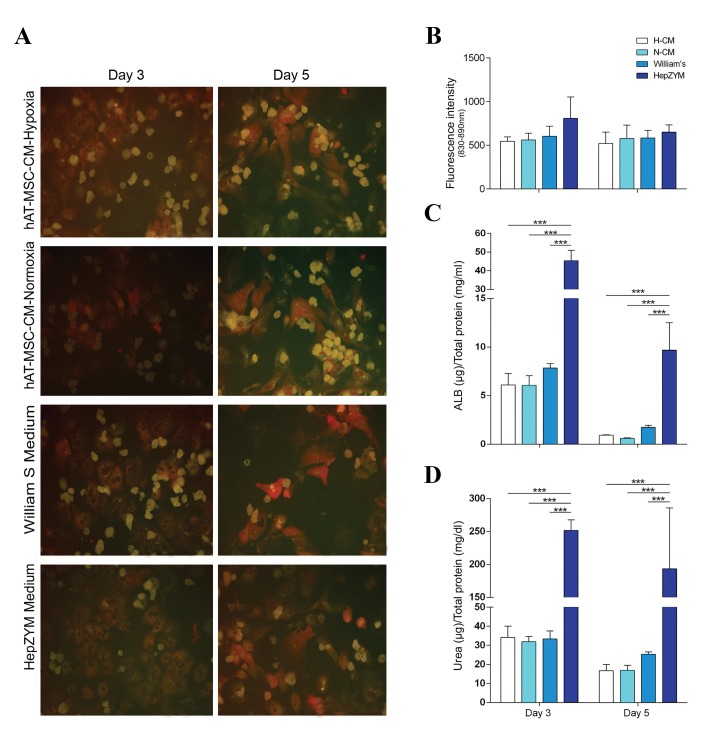
Hepatocyte function analysis in different media. A, B. PROD assay in hepatocytes cultured in different media on days 3 and day 5. Representative image and quantitative analysis of PROD activity in primary hepatocytes. Red areas demonstrated PROD activity in the respective cells. There were no significant differences in the CYP activity between all groups, C. Albumin secretion, and D. Urea synthesis in the different groups. The Alb secretion and urea production from hepatocytes cultured in HepZYM were significantly higher (P=0.0001) on days 3 and 5, compared to the other three groups. The data were presented as mean ± SD (n=5, ***; P<0.0001) (scale bar: 100 µm). hAT-MSC-CM; Human adipose tissue-mesenchymal stromal cells-conditioned media. H-CM; hypoxic-CM, and N-CM; Normoxic-CM.

Cytochrome P450 activity, as a characteristic feature of hepatocyte function, was inspected using the PROD assay. The red areas demonstrated PROD activity in the respective cells (Fig .5A). No significant differences in cytochrome P450 enzyme activity of hepatocytes were seen when fluorescent intensity of cell culture supernatant of all groups compared together (Fig .5B).

### Albumin secretion and urea production

In addition to cytochrome activity, we assessed Alb secretion and urea production of hepatocytes cultured in different groups. The Alb secretion and urea production from hepatocytes cultured in HepZYM were both significantly higher (0.0001) on days 3 and 5, compared to the other three groups (Fig .5C, D). Alb production significantly decreased in all groups on day 5 compared to day 3. We observed no differences in urea production after 3 and 5 days in Hep ZYM.

### Presence of VEGF, HGF and bFGF in human adipose tissue-derived mesenchymal stromal cells-conditioned medium 

We evaluated the range of three major growth factors in both H-CM and N-CM. Further investigations showed higher, but insignificant VEGF expression as a crucial angiogenic factor regulated by hypoxia in H-CM compared to N-CM (P=0.063). In a similar manner, a comparison of both CMs showed no significant differences in bFGF and HGF levels (the data were not shown).

## Discussion

Hepatocytes are considered the best candidates for liver cell therapy. However limitations such as their particularly low proliferation rate and loss of metabolic function during *in vitro* culture (29) have hampered their application. In this study, we treated mouse hepatocytes with CM from hAT-MSCs produced under normoxia or hypoxia conditions. 

Our findings demonstrated that enrichment of culture medium with N-CM or H-CM resulted in higher proliferation in 5-day cultures compared to the hepatocytes cultured in HepZYM media. 

The CM obtained from hAT-MSCs under hypoxic condition remarkably increased glycogen storage of primary hepatocyte after 3 days compared to basic William’s medium or William’s medium supplemented with N-CM, which indicated that H-CM could cause further glycogen storage. In addition, significantly higher glycogen storage levels in HepZYM on day 5 suggested a time-dependent effect of H-CM on glycogen storage. The reduction in PAS^+^ areas in the hepatocytes cultured in HepZYM medium at day 5 compared to day 3 suggested that these cells lost glycogen storage capability even after culture in optimal condition medium.

On the other hand, ICG uptake significantly increased in cells treated with HepZYM and H-CM for 5 days compared to N-CM or William’s medium. No significant difference was found in the ICG uptake levels in cells treated with H-CM or HepZYM. This indicated that secreted factors obtained from hypoxia preconditioning could positively induce ICG uptake at similar levels to HepZYM medium.

Our findings revealed that neither N-CM nor H-CM from hAT-MSCs affected cytochrome P450 enzyme activity levels as well as Alb and urea production.

Hepatocytes could maintain their characteristic functions for only a few days *in vitro* (12). These cells rapidly lose their cuboidal morphology and metabolic functions (29), ultimately leading to cell death. Shulman and Nahmias reported that by using different extracellular matrices (ECMs) such as Matrigel or collagen double- gel configuration enabled them to preserve primary hepatocytes further in vitro (30). In the previous studies were reported that incubation of MSCs under hypoxic conditions for 24 hours did not show any significant changes in the secretome, compared to normoxic conditions (26, 27). The findings of the present study supports this research, which implicated no significant alterations in VEGF, HGF and bFGF expressions under hypoxic conditions compared to normoxic conditions.

It has been reported that overexpression of VEGF in vivo (mouse) leads to increased liver mass, however, this factor only seems to upregulate the hepatocyte proliferation rate in vitro in the presence of sinusoidal endothelial cells (31). Yu et al. (28) showed that conditioning BM-MSCs with 1% hypoxia for 24 hours stimulated VEGF secretion and transplantation of the respective cells into a rat model after partial hepatectomy could moderately improve its condition. In this context, we showed that hAT-MSCs-CM from both hypoxia and normoxia conditions significantly induced higher hepatocyte proliferation rates after 5 days. In contrast, this rate was surprisingly low in hepatocytes treated with HepZYM medium, which is known as a specific medium for hepatocyte culture. Several studies have used HepZYM, as an optimal serum-free medium, for long-term cultures of hepatocytes (32, 33).

It has been shown that co-culturing human hepatocytes with MSCs improves maintenance and function of the hepatocytes (12). This co-culture also leads to stimulation of Alb expression and urea production during 5-25 days of the culture. The improved maintenance of hepatocytes could be related to the trophic factors secreted in MSC CM. According to other research, hepatocytes treated with only MSC-derived factors did not show any improvement in function (34). It has also been stated that co-culture with currently used non-human cells, including mouse embryonic fibroblasts and stromal feeders, could not be an appropriate choice for human hepatocyte culture. In addition, Mallon et al. (35) reported that the latter approach not only had a low efficiency, it also could not be beneficial in the clinic settings due to xenobiotic sources.

In the present study, we used CM obtained from hAT- MSC culture to maintain hepatocyte function in vitro. AT-MSCs could be a suitable candidate for a hepatocyte culture considering their feasibility in isolation and increased numbers of secreted growth factors compared with other sources of MSCs (19). It was shown that no significant difference existed on the levels of growth factors (e.g., VEGF, HGF, and IL-6) secreted from BM- MSCs at 5 or 21% oxygen levels. These growth factor levels were shown to remarkably increase in intensive hypoxic (0.1% oxygen) conditions (26). In another study, Ranganath et al. (36) suggested that time optimization of hypoxia was a crucial factor on paracrine functions of MSCs. Different studies compared the effects of duration of hypoxic conditions (from 16 to 72 hours) on the levels of different secreted factors (27, 34-39). However, an optimum duration and proper pO2 are yet to be found. In the current study, we observed no significant differences in the VEGF, HGF, and bFGF concentrations between the two different CMs. Therefore, further optimization is required for the preconditioning protocol.

In terms of the concentration of various factors in discussed the results, wrote the paper. All authors read hepatocyte cultures, in a previous study van Poll et al. and approved the final manuscript. (16) compared treatment of hepatocytes with 2% and 8% MSC-CM anddemonstrated that cell proliferation References could further be stimulated in hepatocytes treated with 2% MSC-CM compared to those treated with 8% MSCCM. It has also been reported that increased secretome concentrations lead to elevation of IL-6 (40). Taken together, with regards to some variations in the nature of AT-MSCs, further investigations are required to determine the effects of hypoxia on the condition of these cells. In addition, the source of MSC (e.g., adipose, bone-marrow) as well as donor age can definitely change the levels of growth factors in CM. The secretome of MSCs from different donors with different health conditions are not equal (25). In this regard, optimizing a standard protocol can efficiently demonstrate the impact of hypoxia on MSC secretome. 

## Conclusion

The enrichment of William’s basal medium with 4% conditioned media obtained from hAT-MSC under hypoxia improved some hepatocyte physiologic parameters, including viability or proliferation, glycogen storage and ICG uptake in a primary culture. We observed higher hepatocyte viability in those enriched with our CMs compared to the cells cultured in the hepatocyte standard culture medium, HepZYM. Furthermore, H-CM could have superior effects on glycogen storage and ICG uptake of the cultured hepatocytes compared to the N-CM and William’s medium. H-CM had a similar impact in glycogen storage and ICG uptake (at 3rd day and at both 3^rd^ and 5^th^ days, respectively) compared to HepZYM medium. In contrast, the hepatocytes cultured in HepZYM presented better functional maintenance in vitro, as they had higher levels of secretion of Alb and urea production. Further investigations are required to find specific factors secreted in H-CM that lead to improvements in hepatocyte maintenance parameters. Considering that each of HepZYM- and H-CM-treated culture media can individually improve certain hepatocyte parameters, their combination may potentially further preserve in vitro hepatocyte functions. 
