# Conditioned Media Derived from Human Adipose Tissue Mesenchymal Stromal Cells Improves Primary Hepatocyte Maintenance 

**DOI:** 10.22074/cellj.2021.7936

**Published:** 2021-03-01

**Authors:** Zahra Azhdari Tafti, Mehdi Mahmoodi, Mohamad Reza Hajizadeh, Vahid Ezzatizadeh, Hossein Baharvand, Massoud Vosough, Abbas Piryaei

**Affiliations:** 1. Department of Stem Cells and Developmental Biology, Cell Science Research Center, Royan Institute for Stem Cell Biology and Technology, ACECR, Tehran, Iran; 2. Department of Clinical Biochemistry, School of Medicine, Rafsanjan University of Medical Sciences, Rafsanjan, Iran; 3. Molecular Medicine Research Center, Rafsanjan University of Medical Sciences, Rafsanjan, Iran; 4. Department of Medical Genetics, Medical Laboratory Center, Royesh Medical Group, Tehran, Iran; 5. Department of Developmental Biology, University of Science and Culture, Tehran, Iran; 6. Department of Regenerative Biomedicine, Cell Science Research Center, Royan Institute for Stem Cell Biology and Technology, ACECR, Tehran, Iran; 7. Department of Biology and Anatomical Sciences, School of Medicine, Shahid Beheshti University of Medical Sciences, Tehran, Iran; 8. Department of Tissue Engineering and Applied Cell Sciences, School of Advanced Technologies in Medicine, Shahid Beheshti University of Medical Sciences, Tehran, Iran

In this article which was published in Cell J, Vol 20, No 3, Autumn 2018, on pages 377-387, the scale bars in Figures 5-A missed unintentionally during production. The following figure is corrected.

The authors would like to apologies for any inconvenience caused.

**Fig.5:**
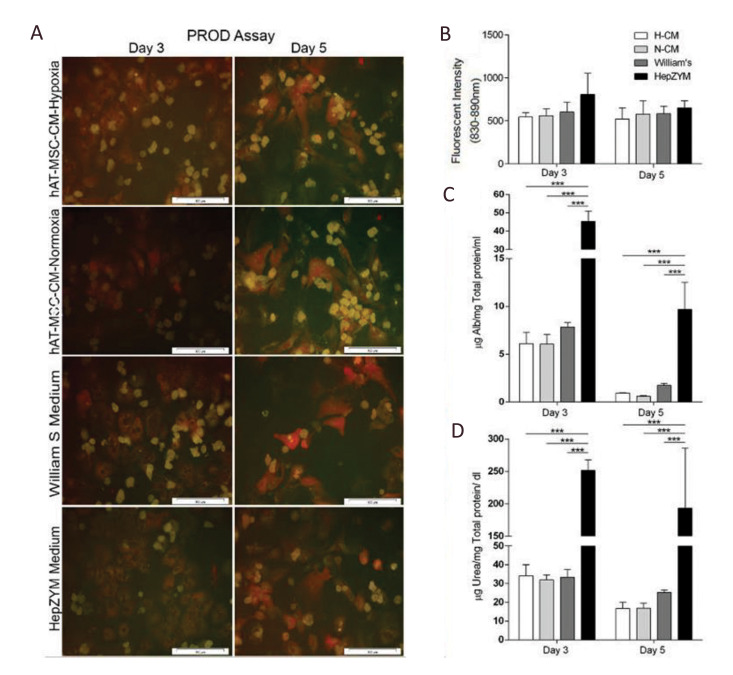
Hepatocyte function analysis in different media. **A, B.** PROD assay in hepatocytes cultured in different media on days 3 and day 5. Representative image and quantitative analysis of PROD activity in primary hepatocytes. Red areas demonstrated PROD activity in the respective cells. There were no significant differences in the CYP activity between all groups, C. Albumin secretion, and D. Urea synthesis in the different groups. The Alb secretion and urea production from hepatocytes cultured in HepZYM were significantly higher (P=0.0001) on days 3 and 5, compared to the other three groups. The data were presented as mean ± SD (n=5, ***; P<0.0001) (scale bar: 100 μm). hAT-MSC-CM; Human adipose tissue-mesenchymal stromal cells-conditioned media, H-CM; hypoxic-CM, and N-CM; Normoxic-CM.

